# Group antenatal care: findings from a pilot randomised controlled trial of REACH Pregnancy Circles

**DOI:** 10.1186/s40814-023-01238-w

**Published:** 2023-03-16

**Authors:** Mary Sawtell, Meg Wiggins, Octavia Wiseman, Anita Mehay, Christine McCourt, Lorna Sweeney, Bethan Hatherall, Tahania Ahmed, Lauren Greenberg, Rachael Hunter, Thomas Hamborg, Sandra Eldridge, Angela Harden

**Affiliations:** 1 Institute of Education, University College London, London, UK; 2 School of Health Sciences, City University of London, London, UK; 3 Department of Health and Human Development, University of East London, London, UK; 4 Wolfson Institute of Population Health, Queen Mary University, London, UK; 5 Department of Primary Care and Public Health, University College London, London, UK

**Keywords:** Group antenatal care, Social deprivation, Limited English proficiency, Pre-specified progression criteria, Feasibility, Process evaluation

## Abstract

**Background:**

Antenatal care has the potential to impact positively on maternal and child outcomes, but traditional models of care in the UK have been shown to have limitations and particularly for those from deprived populations. Group antenatal care is an alternative model to traditional individual care. It combines conventional aspects of antenatal assessment with group discussion and support. Delivery of group antenatal care has been shown to be successful in various countries; there is now a need for a formal trial in the UK.

**Method:**

An individual randomised controlled trial (RCT) of a model of group care (Pregnancy Circles) delivered in NHS settings serving populations with high levels of deprivation and diversity was conducted in an inner London NHS trust. This was an external pilot study for a potential fully powered RCT with integral economic evaluation. The pilot aimed to explore the feasibility of methods for the full trial. Inclusion criteria included pregnant with a due date in a certain range, 16 + years and living within specified geographic areas. Data were analysed for completeness and usability in a full trial; no hypothesis testing for between-group differences in outcome measures was undertaken. Pre-specified progression criteria corresponding to five feasibility measures were set. Additional aims were to assess the utility of our proposed outcome measures and different data collection routes. A process evaluation utilising interviews and observations was conducted.

**Results:**

Seventy-four participants were randomised, two more than the a priori target. Three Pregnancy Circles of eight sessions each were run. Interviews were undertaken with ten pregnant participants, seven midwives and four other stakeholders; two observations of intervention sessions were conducted. Progression criteria were met at sufficient levels for all five measures: available recruitment numbers, recruitment rate, intervention uptake and retention and questionnaire completion rates. Outcome measure assessments showed feasibility and sufficient completion rates; the development of an economic evaluation composite measure of a ‘positive healthy birth’ was initiated.

**Conclusion:**

Our pilot findings indicate that a full RCT would be feasible to conduct with a few adjustments related to recruitment processes, language support, accessibility of intervention premises and outcome assessment.

**Trial registration:**

ISRCTN ISRCTN66925258. Retrospectively registered, 03 April 2017.

## Key messages regarding feasibility

1. What uncertainties existed regarding the feasibility?
  This pilot trial addressed uncertainties around optimum methods for testing the effectiveness of a model of group antenatal care (Pregnancy Circles) in NHS settings serving populations with high levels of socio-economic deprivation and cultural, linguistic and ethnic diversity. This included testing: recruitment methods, uptake of and retention in group care, outcome assessment and approaches to language support. Development of the intervention and feasibility issues around content and delivery, were primarily addressed in previous studies. However, this pilot provided the opportunity to test some remaining uncertainties, including how to maximise retention.
2. What are the key feasibility findings?
  We found that pre-specified criteria for progression to a full trial were all met. While feasibility was established, and most of our processes were fit for purpose, some improvements were identified that would be expected to increase recruitment rates: for example, more flexible recruitment procedures for those wanting a longer time to consider participation and improved communication about certain aspects of the intervention. Ready access to good interpreting options proved key to the recruitment of those with limited English proficiency.
  Other findings included the importance for intervention uptake of minimising the time delay between recruitment and randomisation and an additional contact, by midwives, with participants prior to the intervention commencing. Accessibility of intervention venues was key to uptake and retention as was ensuring all midwives, not just those delivering Pregnancy Circles, were well informed about the intervention and supported women randomised to this arm of the trial in accessing their group care. Retention to the study was impacted by miscarriages and movement out of the study services. For outcome assessment using routine maternity data, utilisation of electronic patient records was more feasible than paper records.
3. What are the implications of the feasibility findings for the design of the main study?
  Our feasibility findings suggest that the trial as piloted was feasible to conduct. The study results indicate that for the full trial a few adjustments would be needed related to recruitment processes, language support, accessibility of premises for intervention delivery, training of maternity service staff about Pregnancy Circles, sample size (to reflect retention to the study) and outcome assessment.

## Background

Antenatal care has the potential to impact positively on maternal, foetal and neonatal outcomes, but limitations to the traditional models of care in the UK have been identified [1]. This is particularly the case for those from socio-economically disadvantaged and minority ethnic groups [2], with this inequity shown to be associated with adverse pregnancy outcomes [3].

Group antenatal care is an alternative model to individual antenatal care. It combines standard clinical midwifery assessment with group discussion, information sharing, self-testing and the opportunity for peer support. Models that have been evaluated vary but usually bring together up to 12 individuals, who live near to each other and have similar estimated dates of delivery, for all their antenatal care facilitated by health professionals (generally midwives). Group antenatal care aims to increase the social, psychological and informational support found to be lacking in traditional antenatal care and to facilitate increased autonomy and empowerment [4]. Models of group care have been implemented in a number of countries and evidence shows that participants view it positively; for example, in the USA [5], UK [6], Australia [7] and Iran [8]. Furthermore, there is some evidence of a positive impact on health outcomes, for example on low birth weight [9], although this is not consistently found [10]. Delivery of group antenatal care with socio-economically disadvantaged and minority ethnic populations has been shown to be successful with evidence of possible added advantages arising from having a mixed group, including with linguistic and ethnic diversity [6].

Systematic reviews highlight the paucity of trials testing group antenatal care and recommend further research [10, 11]. A trial is therefore required to solidify the evidence base for group antenatal care and to test group care in the UK NHS, as opposed to other different health service contexts. This paper reports on an external pilot randomised controlled trial (RCT) testing methods to inform a full UK RCT of a bespoke model of group antenatal care, called Pregnancy Circles [12]. The pilot is part of the larger REACH Pregnancy Programme [13], which is investigating ways to reduce inequalities in access to antenatal care, as well as experience and outcomes of care. Prior to the pilot the REACH team undertook feasibility work to test the intervention in the UK [6, 14].

The principal aim of this pilot trial was to determine optimum methods for testing the effectiveness of group antenatal care in NHS settings serving populations with high levels of socio-economic deprivation and cultural, linguistic and ethnic diversity. This included the assessment of:

1. Methods of recruitment, recruitment rates and reasons for declining participation
2. Uptake of group care
3. Retention in groups and reasons for dropout
4. Data collection for outcome assessment
5. Approaches to language support for delivering the intervention and research activities

Additional aims of the study included:

1. Refinement of the Pregnancy Circles model of group care
2. Assessment of the suitability of our proposed outcome measures
3. Development of a composite measure of a ‘healthy birth’ for the economic evaluation

## Methods

### Study design

The study was an individually randomised controlled trial serving as an external pilot for a fully powered RCT, which was accompanied by pilot process and economic evaluations. Data was collected at baseline and three follow-up points (35 weeks pregnant, delivery of the baby and 4 months postnatal). Participants and maternity staff were unblinded to allocation. Staff supplying outcome information from electronic records and those involved in the analysis of outcomes were blinded until data collection was complete and the analysis plan signed off.

### Randomisation procedure

Women were individually randomised to either the intervention (Pregnancy Circles group antenatal care) or the control group (standard individual antenatal care). The allocation ratio was 1:1. Randomisation was stratified by maternity unit—three were involved in the pilot. Permuted block randomisation with block sizes of *n * = 6 and 4 was used within each stratum; randomisation was carried out once recruitment was complete at each of the three sites. This was conducted remotely by the Pragmatic Clinical Trials Unit (PCTU) at Queen Mary, University of London. Participants were notified verbally and in writing of their trial arm allocation.

### Eligibility criteria

We recruited pregnant people who had booked to have their antenatal care with the maternity services of an inner London NHS trust which serves communities with high levels of socio-economic deprivation as well as cultural, ethnic and linguistic diversity.

Individuals were eligible for participation if they were pregnant and registering for antenatal care at the participating NHS trust maternity services, lived within the geographical catchment areas where Pregnancy Circles were taking place and had an estimated delivery date (EDD) that fitted with those of proposed Pregnancy Circles (women’s EDD had to fall within 2 weeks on either side of the planned 40-week Pregnancy Circles session, based on the date of last menstrual period or 12-week ultrasound scan if available at recruitment). Exclusions included: under 16 years of age, required according to local protocols to be referred to a specialist maternity team for their care (e.g. a ‘vulnerable women’ team), documented as having a learning disability, and unlikely to be able to attend the majority of group sessions.

All those who met the above criteria regardless of their English proficiency were eligible to take part.

### Method of recruitment

In each study area, the participant information sheet (PIS) was mailed to individuals who appeared to meet the inclusion criteria in advance of their first midwife appointment. Recruitment took place at either the first midwife appointment at which individuals register with the maternity service (the ‘booking appointment’—from 8 weeks of pregnancy) or at the first ultrasound scan (at around 12 weeks of pregnancy). Bilingual researchers (where available) or bilingual health advocates (BHA) working locally in the NHS or for an agency, who worked alongside recruiters, provided language support for those with limited English proficiency. If such interpreting was not available, a telephone interpreting service called Language Shop was used [15].

Participants filled in a self-complete baseline questionnaire at the time of consent (see Table 5 for detail on content). Those who were uncertain about participation were given time to consider this at home and recruiters then attempted to follow up. The protocol for this study provides more detail on recruitment [9].

### The intervention

The Pregnancy Circles intervention consisted of eight 2-h antenatal group sessions and one postnatal reunion as outlined in Table 1. The first part of each session involved ‘self-care activities’ (individuals are taught to test their own blood pressure and urine) followed by one-to-one individual health checks with a midwife on a mat in the group space, to one side of the ‘Circle’ while other women had a group discussion facilitated by the second midwife. These clinical checks included: palpation, auscultation and fundal height measurement; discussion about blood pressure, urine, scan and blood results; and questions about foetal movements, personal concerns, mental health and domestic violence. Any general queries were discussed in the group, allowing the individual checks to remain brief (3–5 min). The content of group discussions was expected to be participant-led, supplemented as appropriate by the facilitating midwives to ensure that essential topics were covered, as per national [16] and local trust guidelines (i.e. screening, preparation for labour, safer sleeping, smoking cessation and healthy eating in pregnancy). Participants in the intervention were also invited to a postnatal reunion group held approximately 1 month after the final Circles session.

**Table 1:** The two types of care

Components of type of care	Pregnancy Circles	Standard care
Setting	Community setting	Hospital or community healthcare setting
Number of appointments	8 group antenatal sessions following the NICE primipara schedule [16] (at approximately 16, 25, 28, 31, 34, 36, 38 and 40 weeks gestation) 1 postnatal group reunion 4 weeks after the 40-week appointment Participants receive standard scans, specialist appointments and postnatal care (as per local guidelines)	8 1–1 antenatal clinical appointments (as per NICE primipara schedule) OR 6 1-1 antenatal clinical appointments (as per NICE multipara schedule [16], excluding the 25- and 31-week appointments) Participants receive standard scans, specialist appointments and postnatal care (as per local guidelines)
Clinical checks	Participants check their own blood pressure and urine Palpation, fundal height measurement and auscultation occur during 1–1 time in the group space	Midwives carry out all checks, including blood pressure and urine Palpation, fundal height measurement and auscultation occur during 1–1 appointments in the clinical room
Time spent with the midwife	2-h group sessions (= 16 h). Three to 5 min 1–1 time built into each session	1–1 approximately 20–30-min clinic appointments (= 2 h 20 min – 3 h 30 min)
Type of appointment	Group of 5–12 women due within 4 weeks of each other with 1–1 time built-in Opportunity for peer support	1–1 appointment with the midwife No opportunity for peer support
Continuity of carer	Antenatal continuity from 2 midwives (plus a postnatal reunion), no intrapartum continuity	Traditional clinics, variable antenatal continuity from the midwife, no intrapartum continuity
Parent education	Integrated into the model, personalised to group member's needs through participant-led discussions	Participants signposted to separate parent education sessions. Brief information provided during appointments

In this pilot trial, three Pregnancy Circles were run, one by each of three different participating maternity units in the NHS trust. Teams of two midwives facilitated the Circles, alongside BHA or other support staff as appropriate, offering continuity of care during the antenatal period. The midwives attended a 1-day training course facilitated by the study team. Participants who suffered a pregnancy loss (defined as miscarriage, stillbirth or neonatal death) or other adverse outcomes would be given support as per trust guidelines and would decide what information they wanted the midwives to share with the Circle and whether they wanted to attend the postnatal reunion. The group decided how and when partners were involved in their Circles at the first session.

Anyone who chose to discontinue care in Pregnancy Circles during pregnancy was transferred to the standard care pathway. Midwives followed the trust’s ‘did not attend’ guidelines if needed.

Participants in the control group continued to have standard midwifery care.

### Sample size

The pre-planned sample size was 72, 24 from each of the three maternity units. No formal power calculation for a difference between arms in terms of an outcome measure was undertaken. Rather, the sample size was determined to allow us to assess the assumptions to be used in the sample size calculations for a full trial (e.g. exploring recruitment, uptake and retention rates) with sufficient precision [17]. With a sample size of 72, the width of a 95% confidence interval for a proportion would be at most ± 11.55% which was deemed sufficiently precise.

### Measures used to address study objectives

#### Feasibility measures

To address the primary objectives of this pilot trial, data were collected on a range of pre-specified feasibility measures to inform decisions about progression to a full trial [18] and future trial processes (see Table 2).

**Table 2:** Feasibility measures: assessment criteria, timing of measurement and data collection method

Feasibility measure	Assessment criteria	Timing of measurement	Data collection method
Number available in each catchment	Number of potentially eligible individuals booking for maternity care in the three maternity services	At booking into maternity services	Collected by NHS administrative staff and researchers
Consent to randomisation (recruitment rate)	The proportion of those invited to participate, who consented	At recruitment	Collected by researchers recruiting at the three units
Uptake of intervention	Number assigned to intervention who attended at least one group session	Throughout intervention period	Derived from records kept by the midwives facilitating Pregnancy Circles
Retention in intervention	Total numbers who attended at least 6 of the 8 Pregnancy Circles sessions (or for those who gave birth early, attendance at 75% of the sessions prior to the birth)	End of the intervention period	Reasons for non-take up or non-attendance at certain sessions were gathered via a range of routes including by midwives over the phone when following up non-attendance, in interviews and via hospital records
Questionnaire completion rates	Overall response rate and completion rate for each measure within the questionnaire	Each of the two follow-up questionnaire points	Overall response rate calculated based on non-response after three attempts utilising a range of routes (e.g. email, postal, telephone)

For each of these feasibility measures the levels deemed appropriate to justify progression to a full trial were pre-specified. This pre-specification activity also provided the basis for discussion with the Programme Steering Committee about amendments that would be required (see Table 3). ‘Amber light’ suggests the need for interrogation and proceed with caution and ‘red light’ suggests halting progression unless suitable solutions can be implemented. Additionally, the process evaluation gathered qualitative data about each of these feasibility outcome areas.

**Table 3:** Progression criteria

	**Green light**	**Amber light**	**Red light**
**Recruitment—number available in each catchment** (within the 4-week recruitment window)	Within each catchment area, 60 + pregnant people with appropriate due dates	Within each catchment area, 40–59 pregnant people with appropriate due dates	Within the catchment area, less than 40 pregnant people with appropriate due dates
**Recruitment—percentage who consent to randomisation**	40% who are eligible consent to randomisation	20–40% who are eligible consent to randomisation	Less than 20% who are eligible agree to randomisation
**Uptake of group care model**	8 randomised take up the groups	6–7 take up the group	Fewer than 6 take up the group
**Retention in groups **^**a**^	More than 5 remain in the group for 6 + sessions	4–5 remain in the group for 6 + sessions	Fewer than 4 remain in the group for 6 + sessions
**Follow-up response rate—self-complete outcomes questionnaire(s)**	75% or greater response to follow up	40–74% response to follow up	Less than 40% response to follow up

^a^Allowing for preterm births and moving out of the NHS trust usual catchment area; these were to be analysed as proportion of sessions that could have been attended

#### Proposed participant outcomes for the full trial

In addition to the feasibility measures outlined in Table 2, the pilot trial also aimed to ascertain the suitability of a range of maternal and child health and social outcomes, as well as different routes for obtaining this data (e.g. routine maternity record data transfer and self-complete questionnaires). Summaries of these outcomes are shown in Tables 4 and 5. Views on these were explored within the qualitative process evaluation.

**Table 4:** Proposed outcome measures from maternity record data for the full trial

Outcome category	Description (all to be collected 1 month postnatal)
**Primary**	• Spontaneous vaginal birth (vs caesarean or instrumental delivery)
**Secondary and/or economic evaluation measures**	• Attendance at antenatal care (number attended) • Caesarean delivery (planned, emergency, none) • Epidural/spinal analgesia use in labour • Infant birth weight, defined as low if less than 2500 g • Gestational age at delivery dichotomised as term or preterm (less than 37 weeks) • Breastfeeding initiation (ever initiated)

**Table 5:** Potential measures from self-complete questionnaires for the full trial

Domains	Description of proposed items and standardised measures	Time point		
		Baseline	First follow-up–35 weeks gestation	Second follow-up–4 months postnatal
Socio-demographics	Age Ethnicity (UK Census categories) Main language English speaking ability Parity Education (highest qualification) Type of housing (own home/rented) Occupancy (who live with)	✓		
Social support	Duke-UNC Functional Social Support Questionnaire [19]	✓	✓	
Self-efficacy	Pearlin Mastery Scale [20]	✓		
Prenatal stress	Revised Prenatal Distress Scale [21]	✓	✓	
Empowerment	Pregnancy-related Empowerment Scale (PRES) [22]		✓	
Involvement in decisions about care/satisfaction with care	Included questions from NHS Patient Survey Programme (NPSP), CQC 2017, Picker Institute and new questions designed for this study		✓	✓
Health service usage	Self-reported use of a variety of health services (GP, health visitor, hospital doctor, A&E services, antenatal/postnatal admissions, uptake of immunisations at 2 and 3 months postnatal)		✓	✓
Breastfeeding continuation and exclusivity	Type of milk in first few days after birth/type of milk at 3 months old			✓
Postnatal depression	Edinburgh Postnatal Depression Scale [23]			✓
Postnatal health symptoms	Postnatal symptoms checklist (as used in National Maternity Survey 2010) [24]			✓

### Data collection methods

#### Feasibility data collection

Table 2 shows the timing and methods of collection for the range of feasibility data items.

#### Process data collection—views on trial methods and intervention

Semi-structured, one-to-one interviews were conducted with participants in the Pregnancy Circles and with staff involved in the intervention (midwives, health visitors and interpreters). Some Pregnancy Circles sessions, where women with limited English proficiency were group members, were observed. The aim of interviews and observations was to gain insights into the acceptability of research processes and explore experiences of, and views on, group care.

Process questions relating to the acceptability of trial methods, for example trial arm allocation, were included in follow-up questionnaires (see below). Individuals who declined to take part in the study were offered the opportunity to answer a brief anonymous questionnaire to collect their demographic information and their main reasons for declining.

#### Participant data collection from questionnaires

Self-complete participant questionnaires were provided at three-time points: baseline (at recruitment), first follow-up (35 weeks pregnant) and second follow-up (4 months after the birth). Measures included in the questionnaires are shown in Table 5. An electronic participant-recorded outcome tool (REDCap) provided by the PCTU was developed during the pilot study. Participants were asked at the point of consent for their preference for whether questionnaires were provided to them in paper or electronic format.

The baseline questionnaire was completed at recruitment, after consent and prior to trial arm allocation. It was completed in hard copy format, and an electronic version was tested at the end of recruitment, using a tablet computer.

The expectation in the protocol was that the first follow-up questionnaire would be completed face to face, in the Pregnancy Circles or at standard antenatal care appointments, and that the second follow-up questionnaire, distributed at 4 months postnatal, would be posted to participants. Distribution via an emailed REDCap survey link subsequently became available, and as most participants specified preference for this route, most follow-up questionnaires were delivered in this way. Anyone who requested a hard copy was sent this in the post with a reply envelope.

The research team checked with antenatal clinic staff, prior to contacting participants about follow-up questionnaires, to ensure there were no known reasons why they should not be contacted (e.g. the loss of a pregnancy). Non-responders who had had the questionnaire by email were sent a follow-up email 1 week later, followed by a phone call if there was continued non-response. Anyone who requested discontinuation of Pregnancy Circles care following allocation was asked if they were prepared to continue to provide data for the study.

Individuals with limited English proficiency were supported in completing questionnaires by a BHA or researcher either face to face in clinic, community or home settings or over the phone. Where neither was available, a telephone interpreting service was utilised.

Participants received a £10 voucher if they participated in an interview and for each of the three questionnaires completed.

#### Participant outcome data collection from routine maternity data

Participant outcome data were accessed through patient hospital records (see Table 4) collected through (1) electronic medical records and (2) an audit of paper maternity notes. A comparison of the two types of records, as a check of reliability and data quality, was carried out to determine the best route for accessing the data in a full trial.

#### Economic evaluation data collection

The feasibility of collecting maternity and infant-related healthcare resource use data via the two participant completed follow-up questionnaires and routine maternity data was tested. There is limited evidence on the validity and suitability of a measure of generic health-related quality of life such as the EuroQol EQ-5D in pregnancy [25]. A current preference-based patient-reported outcome measure that could be used in an economic evaluation of a pregnancy intervention could not be found. A rapid review of the literature for positive birth outcomes and maternity preference-based outcomes was therefore carried out by two researchers on the study. A patient and public involvement (PPI) group was presented with items identified in the review to stimulate discussion on views on the definition of a ‘healthy and positive birth’ for use in a future preference-based outcome measure.

### Data analysis methods

#### Analysis of outcome data

Data were analysed for completeness and for usability in a full trial using descriptive statistics. No hypothesis testing for between-group differences in participant outcome measures was undertaken. Outcomes were summarised in each arm using mean and standard deviation for continuous data and number and percentages for categorical data.

#### Process evaluation analysis

Thematic analysis of the qualitative data was carried out. Interview audio records and observation notes were transcribed, and the data were coded using the software package NVivo 11. Themes were generated through a process of iterative coding, with team members discussing the findings.

#### Economic evaluation analysis

Descriptive statistics (mean, standard deviation and proportion of missing data) were reported for healthcare resource use. The cost of the Circles was calculated using data collected on the number of individuals per Circle, the frequency of Circles and information collected as part of the process evaluation on the amount of time midwives spent preparing for and delivering the circles. Midwife time was costed as a NHS Band 6 nurse (£47 per hour including overheads) [26].

## Results

### Recruitment

In line with the first objective and progression criteria (see Table 3), the results will be discussed in relation to various aspects of recruitment to the pilot trial.

#### Participant numbers

**Fig. 1:**
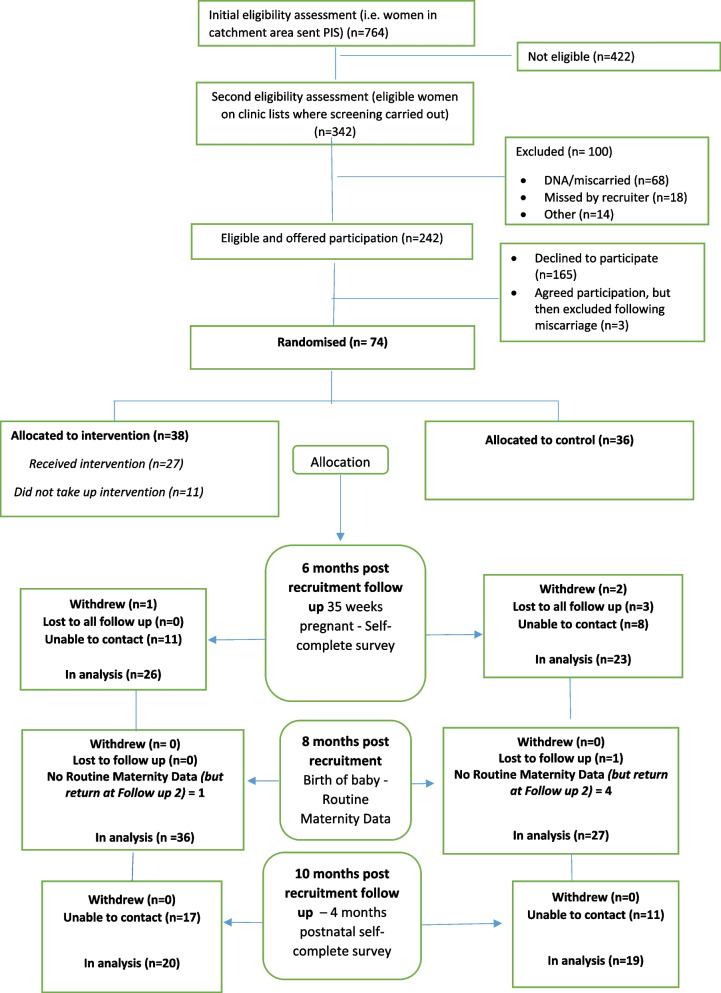
Reach Pregnancy Circles pilot trial—CONSORT diagram

In this pilot study, 74 were randomised as study participants (see Fig. 1), two more than the a priori randomisation target.

For the process evaluation, interviews were undertaken with participants (*n* = 10), facilitating midwives (*n* = 7) and other stakeholders (*n* = 4), from across the sites. Two observations of Pregnancy Circles sessions were undertaken.

#### Numbers eligible

The participant information sheet was mailed to 764 individuals, who had been referred for antenatal care at the three maternity units. They all lived in the target catchment areas and appeared to have an appropriate due date.

Patient lists for booking or scan clinics where recruitment took place were screened and identified 342 people who were likely to be eligible for the study; numbers per catchment area are shown in Table 6. The number subsequently offered trial participation was 242. Reasons for not offering participation included (1) miscarriage during the period between referral into the service and clinic attendance and (2) the recruiter missing the person in the clinic and at subsequent follow-up attempts.

**Table 6:** Feasibility outcomes—results

Feasibility outcome	Total
**Recruitment—number of available pregnant individuals in each catchment** **(appeared eligible at first screen and sent PIS) (*n*/*N*) **^**a**^	764
Catchment 1	238/764 (31%)
Catchment 2	135/764 (18%)
Catchment 3	391/764 (51%)
**Recruitment—number of eligible individuals on the clinic list where recruitment took place—(second screen)** (*n*/*N*)^**a**^	342
Catchment 1	117/342 (34%)
Catchment 2	85/342 (25%)
Catchment 3	141/342 (41%)
**Recruitment—percentage randomised (number consenting to randomisation by number offered trial participation (*n*/*N* (%)))**^**a**^	77/242 (32%)
**Uptake of group care model (number attending at least on session of group care model/number randomised to group care (%))**^**a**^	27/37 (73%)
**Retention in groups**^**a**^	
Number of sessions attended (number attended/number of sessions offered (%))^c^	141/296 (48%)
Number of possible sessions attended (allowing for early delivery/moving out of area) (number sessions attended/number of possible sessions to attend (%))	141/256 (55%)
Participants who attended 6 + sessions (number attending 6 + sessions/number randomised to group care(%))	13/37 (35%)
Participants who stayed for 75% + of possible sessions, allowing for early delivery/moving out of area (number who stayed for 75%/number randomised to group care (%))	18/37 (49%)
Median number of sessions attended in Pregnancy Circles arm (median)	4^b^
**Follow-up response rate—self-complete outcome questionnaire(s)**^**a**^	
Follow-up 1 (number responding/number of randomised participants (%))	49/74 (66%)
Follow-up 2 (number responding/number of randomised participants(%))	39/74 (53%)

^a^Progression criteria to full trial

^b^Non-attenders were followed up to ensure that they received adequate antenatal care

^c^Based on each woman being able to attend all 8 standard care sessions

The numbers of eligible individuals ranged between 85 and 141 across the three areas; the progression criterion of ‘numbers of available individuals in each catchment area’ for recruitment was met at the green light level (i.e. 60 + ; see Table 3).

#### Percentage who consented to randomisation

This pilot trial had a 32% recruitment rate (see Fig. 1 and Table 6). Interviews with participants indicated that the recruitment was particularly boosted by the novelty of the intervention and by the possibility of greater social support.

I was really interested to participate in it because I thought it would be different to normal.

It would be a chance to meet other women, I thought that was brilliant because I didn’t do NCT [private pregnancy classes]. 

All 165 people who declined to participate were invited to take part in a nested study to explore factors behind their decision; 73 took part in this nested study. The main reason for non-participation was not having enough time to attend the 2-h sessions (66%): getting time off work and childcare responsibilities were frequently mentioned reasons. Some who declined specified they would have consented if a creche had been offered or if children could attend the session. Other common concerns were practical, such as the location or the timing of the proposed groups and health issues. The model had less appeal to those who felt well supported and although only one person declined citing language problems, a quarter of decliners had ‘little’ or ‘no’ English. Reasons given by the latter included childcare, getting time off work and complex pregnancies.

Only four of 73 who responded to the decliner questions said they did not like the idea of being in a group or had concerns about privacy. This was an unexpected finding as stakeholders were concerned before implementation about clinical checks in the group space. We discovered that a few individuals did not understand the randomisation process fully.

I thought it was a case where with every [pregnancy circles] session, you were going to get picked whether you are going to the group or whether you are having the one-to-one.

The recruitment rate of 32% was within the amber category of our traffic light criteria for progression to the full trial (20–40%; see Table 3). Areas for improvement that could be expected would raise this rate were identified, such as clearer communication to minimise misunderstanding, provision of childcare at sessions and even greater flexibility for those requiring a longer time to consider participation [27].

#### Participant characteristics—age, parity, language, education—intervention and control

The characteristics of the 74 pilot trial participants are detailed in Table 7. They were predominantly over the age of 25 (78%) and of either Asian (45%) or white (38%) ethnicity. On the baseline questionnaire, 15% of participants said that they did not speak any English or spoke it ‘Not well’. A large majority (85%) lived in rented accommodation, and three-quarters were living with their partner/husband. A third of the participants (34%) had no education beyond GCSE or vocational level. Just under half (47%) had already had a child.

**Table 7:** Participant characteristics

Characteristic	Control arm *n*/*N* (%)	Intervention arm *n*/*N* (%)	Total *n*/*N* (%)
Age: over 25	29/36 (80.56)	30/38 (78.95)	59/74 (78.38)
Ethnicity: White	15/36 (41.67)	13/38 (34.21)	28/74 (37.84)
Ethnicity: Black or Black British	5/36 (13.89)	2/38 (5.26)	7/74 (9.46)
Ethnicity: Asian or Asian British	15/36 (41.67)	18/38 (47.34)	33/74 (44.59)
Ethnicity: mixed/other	1/36 (2.78)	4/38 (10.53)	5/74 (6.76)
Language: English as the main language	24/36 (66.67)	16/38 (42.11)	40/74 (54.05)
Language: speaks English ‘well/very well’	34/36 (94.44)	28/38 (75.11)	62/74 (83.78)
Language: speaks English ‘Not well’	2/36 (5.56)	8/38 (21.1)	10/74 (13.51)
Language: ‘I do not speak any English’	0/36 (0.00)	1/38 (2.63)	1/74 (1.35)
Parity: has had 1 or more child	16/36 (44.44)	19/38 (50.00)	35/74 (47.30)
Education: no qualifications	1/36 (2.78)	2/38 (5.26)	3/74 (4.05)
Education: GCSE and vocational	12/36 (33.33)	10/38 (26.32)	22/74 (29.73)
Education A level or equivalent	4/36 (11.11)	6/38 (15.79)	10/74 (13.51)
Education: degree/postgrad	19/36 (52.78)	20/38 (52.63)	39/74 (52.70)
Tenancy: rented-council/housing association/private	29/36 (80.56)	34/38 (89.47)	63/74 (85.14)
Tenancy: own property	4/36 (11.11)	4/38 (10.53)	8/74 (10.81)
Tenancy: temporary accommodation	0/36 (0.00)	0/38 (0.00)	0/74 (0.00)
Adults live with married/partner	27/36 (75.00)	28/38 (73.68)	55/74 (74.32)
Adults live with other adults	3/36 (8.33)	1/38 (2.63)	4/74 (5.41)
Adults live with no other adults	1/36 (2.78)	2/38 (5.26)	3/74 (4.05)

In this small sample, the arms were fairly well balanced on these characteristics, with the notable exception being the higher proportion of individuals in the intervention arm identifying as speaking English ‘not well’ or ‘not at all’ (24% intervention vs 6% control).

### Uptake and retention

#### Intervention uptake

Thirty-eight participants were randomised to the treatment arm. Participants in the treatment arm were more positive about their allocation than those in the control arm (follow-up 1: 69% vs 56%). One participant miscarried following randomisation and prior to the commencement of the Pregnancy Circles. Of the 37 remaining, 27 (73%) took up the intervention, by attending at least one group session (see Table 6). The uptake level was similar across the three pilot sites (range 69–77%).

There were several reasons provided for the lack of uptake of the intervention. The separation of participant consent and randomisation, with the former carried out at the booking or scan appointment and the latter as a block once recruitment to a specific Circle had taken place, appeared a barrier to intervention uptake. This separation, which for some participants was as much as 4 weeks, impacted on the understanding of, and motivation for, study involvement by some recruits. Related to this was that some participants told their midwives that they had changed their minds and now wanted one-to-one care. The process data suggests that sometimes hospital midwives encouraged this switch back to the known standard care as opposed to the ‘unknown’ group care intervention. In one pilot site, where finding an appropriate venue for the Pregnancy Circles to be held had proved challenging, the venue used was relatively distant from the hospital and people’s homes. Despite this being explained during the consent process, three who did not take up the intervention gave distance as the reason when contacted by midwives.

The uptake level was within the green traffic light category (see Table 3). Although our recruitment numbers varied slightly above and below the exact 12 we initially planned for, we had eight or more take up the intervention in each site.

#### Retention within the intervention

In all three pilot sites, the Pregnancy Circles intervention was delivered to the expected timetable, with the intended eight antenatal sessions offered in each site. Of the 37 individuals able to take up the intervention, 13 (37%) attended six or more of the eight scheduled sessions (i.e. 75% of sessions). As anticipated (and pre-specified), some non-attendance at sessions was inevitable for reasons such as early delivery or moving out of London. When these unavoidable reasons are taken into account, 18 (49%) attended 75% or more of the sessions that they could have attended (e.g. before they delivered early or moved away). As is standard in the NHS, individuals who did not attend their antenatal appointment (whether the group session or individual appointment) were followed up by a midwife to ensure they received adequate antenatal care, as per local guidelines.

The pattern of attendance suggests that missed sessions occurred across the spread of sessions for the intervention; often these were for illness, work or childcare conflicts, and the participants returned for further sessions.

There was a mixed picture across the three pilot groups regarding retention, with one site in the red light zone (< 4 attending 6 + sessions; see Table 3), one in the amber and one in the green. Our pre-specified criteria allowed for retention rates to take into consideration early delivery or movement out of the area. With this allowance, two sites had the amber light and one had the green light for retention rates (see Table 8).

**Table 8:** Retention: variation by site

Site	Attended 6 + sessions *n*/*N* (%)	Attended 75% + of the sessions *that they could have attended* (allowing for early delivery/moving) *n*/*N* (%)
1	2/11 (18%)	4/11 (36%)
2	4/13 (31%)	5/13 (39%)
3	7/13 (54%)	9/13 (69%)

Whereas some missing of individual sessions occurred, when a participant chose to leave the intervention, a key reason was the development of additional health concerns or vulnerability within the pregnancy. While individuals who became high risk were able to continue in their Pregnancy Circles while also receiving additional specialist care, this was not always understood by hospital midwives who were not directly involved in Pregnancy Circles. This was illustrated by a Pregnancy Circles midwife interviewee.

[The hospital midwives say] ‘I am booking you an appointment to come and see me here’ and [the women] are saying, ‘Yes, but I have [ Pregnancy Circle care]…’ and they were saying ‘No, no, no that’s not real’. And that problem comes from management because if the management were supporting us they would know. 

While the key reasons for leaving the intervention were miscarriage, moving out of the area or escalating health concerns, there were a small number who left the intervention and returned to 1 to 1 care, where the research team were unable to ascertain reasons. Midwife interviewees suggested that accessibility of intervention venues was a key factor facilitating retention.

### Outcome measures

#### Questionnaire completion rates

Two-thirds of the randomised participants across both arms completed the first follow-up questionnaire (66%, *n* = 49); at the second follow-up, this fell to 53% (*n* = 39) (see Table 6).

As highlighted in the CONSORT diagram (Fig. 1), the response rates were similar by arm of the trial at both time points: completed follow-up 1: 68% (*n* = 26) intervention, 64% (*n* = 23) control; follow-up 2 completed: intervention 54% (*n* = 20), control 53% (*n* = 19). Of the 10 participants who did not ever take up the intervention, four still completed follow-up questionnaires. Our follow-up rates for the questionnaires fell within the amber traffic light category of the progression criteria (which we defined as 40–74% response to follow-up) (see Table 3).

#### Assessment of outcome measures for the full trial

We received birth data from maternity electronic records for 63 (89%) of the participants who had not withdrawn from the study. The intended comparison of this data gathered from electronic records with paper record data gathered by a member of the research team proved less straightforward than expected. In one site, paper records were no longer used; in another, these records were moved to an off-site storage facility soon after the birth and were then hard to retrieve. In order to explore how valid and trustworthy the transferred electronic data was as a source of outcome data, we proceeded with a modified comparison of data sources. This took the form of a local research midwife extracting data from paper records where they were accessible and entering these into a spreadsheet that was uploaded to the PCTU. The study statistician then met with the research midwife and wider study team to interrogate the data from the two data sources. This exercise showed that the data collected from both sources had high completion and was sufficiently similar (in terms of missing data and responses) on most outcomes and that the process was significantly more robust and straightforward when utilising the electronic records. The discrepancies primarily revolved around different interpretations of specific outcomes. This exercise thus provided additional learning around the definitions and instructions that would need to be utilised to garner the most accurate electronic data transfer from a range of trust site data teams in a full trial. For instance, the comparison highlighted different interpretations of what constituted a ‘spontaneous’ birth or different risk categories in pregnancy. With suggested clarifications to the definition, the primary outcome of spontaneous vaginal birth was agreed to be feasible for the full trial.

The completion of secondary outcome measures was also assessed as part of the pilot trial. Table 9 shows that completion rates were high, with all measures being completed by over 90% of the respondents. Additionally during the pilot, we had input from our Programme Steering Committee members, from development work on our survey tools with user groups (including language interpreters) and from subsequent work on outcomes, drawing on a systematic review of literature that was being conducted alongside this pilot trial. Some revision of the list of secondary outcome measures proposed for the full trial was made as a result.

**Table 9:** Completion rates on individual outcomes from returned self-complete questionnaires

**Domain**	**Outcome measure**	% responded to this question
Social support	Duke UNC Functional Social Support Scale	95%
Self-efficacy	Pearlin Mastery Scale	100%
Prenatal stress	Revised Prenatal Distress Questionnaire	100%
Empowerment	Pregnancy-related Empowerment Scale (PRES)	100%
Involvement in decisions about care/satisfaction with care	Included questions from NHS Patient Survey Programme (NPSP), CQC 2017, Picker Institute and new questions designed for this study	Various questions: all between 94 and 98%
Health service usage	Self-reported use of a variety of health services (GP, health visitor, hospital doctor, A&E services, antenatal/postnatal admissions, uptake of immunisations at 2 and 3 months postnatal)	94%
Postnatal depression	Edinburgh Postnatal Depression Score	100%
Postnatal health symptoms	Postnatal symptoms checklist	100%

### Economic evaluation

As part of the rapid literature review for developing a preference-based outcome, the following areas were identified as key priorities for individuals in having a positive birth: mother’s own health and well-being pre- and postnatal and during the birth, health of the baby, role of their partner and family in the pregnancy and birth, early antenatal care, what they want to happen during the delivery, continuity of care and housing and financial security. Individuals who were part of the Pregnancy Circles were asked to join the PPI forum. Five mothers and their babies attended the PPI session along with a health visitor who had been involved in the Pregnancy Circles. Following the discussion with the PPI forum, we identified the following priorities:

- Healthy mother
- Healthy baby
- The information provided to pregnant individuals including how medical professionals communicate with them
- Choice and feeling in control
- Feeling supported/empowerment
- Continuity of care with midwife and health visitor
- Infant feeding

Individuals were also asked to rank these in terms of what was most to least important to them and found this possible to do. Information on all of these priorities is being collected as part of the full trial.

#### Cost of pregnancy circles in this pilot trial

The costing processes were tested within this pilot. Midwives found that each intervention session took 4 h in total with 1 h either side of the 2-h Circle to set up and pack up with generally two midwives per Circle. There were five to 11 participants per Circle, although midwives found that they needed to do additional work outside the Circles to either record and follow up on test results, as well as additional appointments for those who were unable to attend a circle. Assuming eight participants per Circle, eight Circles in total and an additional 15 min per individual outside the circle, the cost for all eight Circles was £3760, £470 per participant. For the upper estimate of the cost per woman, assuming five participants and three midwives per Circle (a model that was used occasionally) and 25 min outside the Circle for each individual, the cost for all Circles was £5302, £1060 per participant. For the lowest estimate, assuming 11 participants per Circle and only half needing 15 min each outside of the Circle and that set-up and pack-up times were a total of 1 h, the total cost for the circles was £2773 or £252 per participant. For individuals receiving standard midwife care, the cost of eight appointments would be £226, assuming a total of 40 min work per appointment. The follow-up questionnaire items related to service use were completed less well than others, but were considered sufficiently robust for the main trial, with minor amendments to instructions.

## Discussion

This pilot trial achieved its primary objective of determining robust methods for testing the effectiveness of group-based antenatal care in an NHS setting serving populations with high levels of socio-economic deprivation and cultural, linguistic and ethnic diversity. The five progression criteria, which corresponded to aims to test the feasibility of different aspects of our methods and were pre-specified at three traffic light levels, were all met at either green or amber levels. This suggested that our methods were appropriate and that advancement to a full trial was acceptable.

The use of these progression criteria facilitated detailed, systematic and transparent decision-making about next steps at the end of the study [18]. It also highlighted specific areas of vulnerability in our processes, allowing us to develop improvements both during the pilot and subsequently for a full trial. For example, retention to the intervention was achieved at the amber level but varied substantially across the three areas. Process evaluation data suggested that good communication about the intervention and accessibility of intervention venues were key factors facilitating retention. Other studies of group interventions in community settings have described the importance of aspects of premises, including accessibility [28]. Planning for the full trial intervention incorporated these findings.

This pilot had some success in recruiting an ethnically and linguistically diverse sample. Over half of the sample came from ethnic minority groups, and approximately 15% had limited English proficiency. While the specific aim of exploring language support options for the intervention and research processes was met (these aspects will be covered in a separate paper), there was not a progression criterion for language support or diversity of participants. In hindsight, this was a limitation—a pre-set criterion around recruitment, retention and follow-up of those with limited English proficiency and also other socially disadvantaged participants would have provided even greater focus. Further reflections have led to a commitment to explore in more depth, in the main trial, how different trial and intervention processes are impacted both by limited English proficiency and minority ethnic group status, including clarifying the midwives’ ability to access face-to-face interpreters during Pregnancy Circles.

Additional aims were to assess the utility of the proposed outcome measures for the main trial and the economic evaluation. The assessment of the suitability of the primary outcome measure (spontaneous vaginal birth) was achieved. Furthermore, the development for the economic evaluation of a composite measure of a ‘positive healthy birth’ was initiated with the identification of what individuals think is important in order to achieve a positive pregnancy, birth and postnatal experience. This has led to a discrete choice experiment that is underway so that the identified priorities can be weighted for use in the full trial economic evaluation. Calculation of the pilot trial intervention costs allowed our economic evaluation processes to be tested. The specific costs produced apply only to the context in which the intervention was delivered in the pilot. The economic evaluation for our full trial can be expected to yield more generalisable economic findings for group antenatal care delivered in the NHS.

The intention had been to run the full trial in the same single NHS trust where the pilot was conducted. In fact, the full trial is now running in multiple NHS trusts both in and outside of London [29]. This was because during the pilot it was recognised that it would not be possible to incorporate Pregnancy Circles within a single maternity service at the scale required to run the full trial. The learning from the pilot has been invaluable in directing our work with different local research teams and study populations, to make the conduct of the full trial as efficient and effective as possible.

## Data Availability

The datasets analysed in the study are available from the last author, Angela Harden, who can be contacted via the corresponding author, upon reasonable request.
